# Investigation of Dosimetric Evaluation and Treatment Planning Time of Inverse Planning Optimization in Combined Intracavitary and Interstitial Brachytherapy for Cervical Cancer

**DOI:** 10.7759/cureus.83330

**Published:** 2025-05-01

**Authors:** Yoshifumi Oku, Souichirou Itou, Shigeyoshi Wakamatsu, Yuushi Niiyama, Masahiko Toyota

**Affiliations:** 1 Division of Radiology, Kagoshima University Hospital, Kagoshima, JPN; 2 Department of Radiology, Kagoshima University Graduate School of Medical and Dental Sciences, Kagoshima, JPN

**Keywords:** cervical cancer, hipo, inverse planning, ipsa, optimization

## Abstract

Clinical evidence demonstrating the effectiveness of optimization and efficiency of treatment plan is limited because the Inverse planning optimization of source position and dwell time variations is complex. Our purpose was to investigate the comparison of the dosimetric evaluations and treatment planning time in two inverse planning algorithms with the conventional Manchester treatment planning for cervical cancer brachytherapy. We retrospectively identified 14 patients who underwent manually and inversely optimized treatment plans using inverse planning simulated annealing (IPSA) and hybrid inverse planning optimization (HIPO). The analysis was performed to analyze the effects of various factors on the dosimetric evaluation indices, such as the D90 for the high-risk clinical target volume (HR-CTV) and D2cc of the organ at risk (OAR), and the distribution of dwell time and optimization time in each algorithm. In most plans, D90 of the HR-CTV exceeded 7 Gy, and the D2cc of the OARs, on average, was below the tolerance dose for all plans. However, the HR-CTV D90 and D2cc of the IPSA-optimized treatment plan tended to be smaller than those of the other plans when the dwell time deviation constraint value of the optimization parameters was increased. The treatment plans used in the Manchester method and those obtained by IPSA and HIPO have similar dose distributions and dose volume histogram parameters. Moreover, the time required to create a treatment plan was reduced by the IPSA and HIPO. Also, it was suggested that IPSA may result in extreme source dwell positions and dwell times.

## Introduction

Brachytherapy (BT) has entered an era in which multimodality images, such as computed tomography (CT) and magnetic resonance (MR) images, are used to determine the three-dimensional region of the target and at-risk organs and optimize the administered dose distribution [1-3]. If a tumor is large and irregularly shaped, there are cases where standard intracavitary irradiation using tandem ovoids may result in insufficient dose to the tumor [4,5]. For such cases, a combined intracavitary BT (ICBT) and interstitial BT (ISBT) is recommended, and it is actively implemented in our hospital. IC/IS-BT is a more complex treatment method than ICBT, and it often takes time to optimize the puncture technique and dose distribution; an optimization procedure has not been established [6].

Recently, the two inverse planning methods available are inverse planning simulated annealing (IPSA) [7-11] and hybrid inverse planning optimization (HIPO) [12-14]. In BT for cervical cancer, the clinical application of inverse planning is still not widespread because of the limitations of catheter size and placement, and the small number of catheters compared to BT for prostate cancer. Radiation oncologists must rapidly determine the optimal treatment plan. Although various dosimetric evaluations can be used for plan comparison, they become complicated when treatment plans in a short period of time are analyzed. The clinical decisions of treatment planning made by radiation oncologists are time-consuming and based on subjective assessments of the planned dose distributions, considering only the most important dose distribution of the plan. Cunha et al. analyzed the comparison with traditional optimization methods, such as Manchester. Inverse planning techniques may lead to large variations in the magnitude of time at adjacent dwell positions. Also, a large variance in intracatheter dwell times may lead to elevated doses proximal to the dwell position, with a relatively long dwell time in the target and organ at risk (OAR) [15]. In contrast, the inverse planning optimization algorithm has short treatment planning time, higher target coverage, and a lower dose to the OAR than planning [16]. However, because inverse planning techniques do not focus solely on the IC/IS-BT and require expertise, their use is limited. Therefore, the present study aimed to compare three methods, the IPSA algorithm, the HIPO algorithm, and HIPO + sampling, for cervical cancer BT, and examine their clinical usefulness.

## Materials and methods

Patient characteristics

For the inverse planning optimization algorithm, fourteen consecutive patients with cervical cancer who underwent IS/IC-BT using the microSelectron HDR system (Elekta AB, Stockholm, Sweden) were included. The treatment was administered in three or four fractions. Detailed patient information is presented in Table 1. The Institutional Review Board (IRB) of our university hospital approved this retrospective study (No. 230071). Squamous cell carcinoma and adenocarcinoma are the predominant histological types of uterine cervical carcinoma.

**Table 1:** Patient characteristics for 14 cases used in this study.

Characteristics		Median	Range	Total (n)
Patients' age (years)		61	24-71	14
Histologic type	Squamous cell carcinoma			13
	Adenocarcinoma			1
FIGO stage	II A			2
	II B			2
	Ⅲ A			1
	Ⅲ B			3
	Ⅲ C			4
	Ⅳ A			1

The IC/IS-BT treatment plan used in this study was created as follows: The Venezia applicator (Elekta AB) was used for intracavitary irradiation, and a 294 mm stainless steel needle (hereinafter referred to as “needle”) (Elekta AB) was used as the applicator for interstitial irradiation. After the Venezia applicator was inserted, an additional needle was inserted into the area where the dose was expected to be insufficient.

Comparison method

Based on the actual implementation of the IC/IS-BT treatment plan, we retrospectively created a treatment plan that optimized the dose distribution using the Manchester method and IPSA, HIPO, and HIPO + sampling points during BT. We compared the treatment plans used in the Manchester method with those optimized using the IPSA, HIPO, and HIPO + sampling points.

Image Acquisition

All patients, in the lithotomy position and after implantation and insertion of the IC/IS-BT applicators and the interstitial needles inserted in their bodies, were scanned using a CT scanner (SOMATOM Sensation; Siemens, Germany) with a slice thickness of 2.0 mm. Image data were transferred to a treatment planning system (OncentraBrachy version 4.6.2; Nucletron, Elekta Company, Netherlands).

Treatment Planning

A radiation oncologist manually delineated the contours of the cervix and uterus on the daily fusion images, according to the recommendations of the Groupe Européen de Curiethérapie (GEC) and the European Society for Radiotherapy & Oncology (ESTRO) - GEC-ESTRO Committee [17-19] and Japanese Society for Radiation Oncology Consensus Guidelines of combined intracavitary and interstitial BT for gynecological cancers [20].

**Figure 1:**
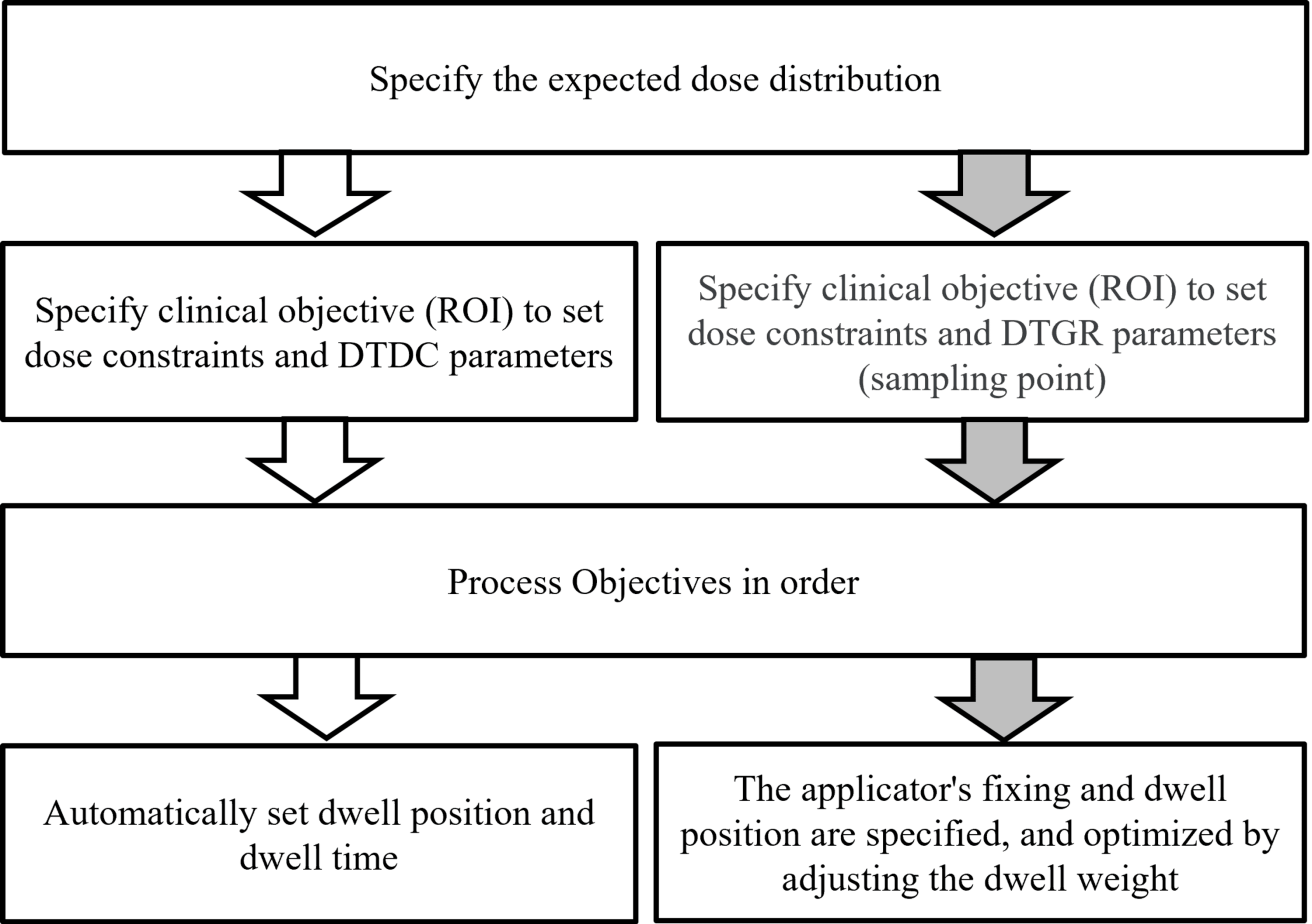
Overall scheme of the workflow for the inverse planning optimization.

The target dose values for BT for cervical cancer at our hospital were a high-risk clinical target volume (HR-CTV) D90 of 7 Gy or more, rectum and small bowel D2cc of 6 Gy or less, and bladder D2cc of 7 Gy or less. First, the Manchester method was based on the IC/IS-BT treatment plan in which irradiation was performed. Figure 1 shows a diagram of the Inverse planning procedure used in this study. In the optimization using IPSA and HIPO, the minimum dose (minimum value), maximum dose (maximum value), and relative weight (weight) were set as parameters for each contour. In addition, the setting value for dwell time deviation constraint (DTDC) and dwell time and dwell time gradient restriction (DTGR) was set to 0.0-1.0. DTDC adjusts the variation of dwell time, while DTGR adjusts the variation of dwell time gradient. In this study, the dose constraint parameters were set to a minimum HR-CTV dose of 7 Gy based on the target dose described above. In addition, the maximum dose for OARs was set by considering the average D2cc dose in the IC/IS-BT treatment plan used in clinical practice and the target dose mentioned above. Normal tissue is a dose constraint for tissues that are not depicted as an outline. This parameter minimizes the dose delivered to tissues. The weight of the maximum dose to the uterus, HR-CTV, and normal tissue was set low to avoid affecting the overall dose distribution.

IPSA Planning 

IPSA is an optimization calculation based on objective variables, with each dwell time as a variable. This is an algorithm that specifies target doses for multiple regions of interest (ROIs) (targets and OARs) and calculates source placement and dwell time.

The optimization can be performed immediately after contouring and applicator reconstruction. Table 2 lists the initial optimization settings used in this study. The HR-CTV was used as the reference target. The minimum surface/volume dose (7 Gy) was set higher than the prescribed dose during optimization to increase the coverage while maintaining the dose to OARs at a constant level. During optimization, the dose of 90% of the HR-CTV D90 was normalized to 100% of the prescribed dose (7 Gy). The optimization parameters were set, and the minimum dose (minimum value), maximum dose (maximum value), and relative weight (weight) were set as parameters for each contour. In addition, the initial setting value of DTDC=0.0-1.0 was used for DTDC, which adjusts the variation of dwell time.

**Table 2:** Dose-volume objectives used for the IPSA plans. HR-CTV: high-risk clinical target volume, DTDC: dwell time deviation constraint, IPSA: inverse planning simulated annealing

Contour	Min (Gy)	Weight	Max (Gy)	Weight
HR-CTV (surface)	7	100	14	10
Bladder (surface)			4.5	50
Rectum (surface)			3.5	30
Sigmoid (surface)			3.5	30
Bowel (surface)			3.5	30
DTDC	0.0 – 1.0			

HIPO Planning

HIPO can adjust the source dwell time using the source dwell position set by the user in advance to meet the dose conditions of the specified ROI (target and OAR). The source dwell positions were set as those for Manchester method. The optimization parameters are listed in Table 3. Similar to the DTDC, the DTGR is a modulation restriction parameter for HIPO that restricts large fluctuations between dwell times in neighboring dwell positions. It is also a relative value between 0.0 and 1.0, the higher the value, the smaller the fluctuation. Moreover, the HIPO enables manual control of the sampling point settings for ROIs. For a high optimization precision, we increased the number of sampling points in proportion to the volumes of the targets and OARs.

**Table 3:** Dose-volume objectives used for the HIPO plans. HR-CTV: high-risk clinical target volume, DTGR: dwell time gradient restriction, HIPO: hybrid inverse planning optimization

Contour	Min (Gy)	Weight	Max (Gy)	Weight
HR-CTV	7	100	14	10
Bladder			4.5	50
Rectum			3.5	30
Sigmoid			3.5	30
Bowel			3.5	30
Normal tissue			14	0.1
DTGR	0.0 – 1.0			

Next, we used the HIPO in the sampling point set. The optimization parameters are listed in Table 4. Thus, the number of sampling points changed according to the different volumes of the targets and OARs.

**Table 4:** Dose-volume objectives used for the sampling points in HIPO plans. HR-CTV: high-risk clinical target volume, DTGR: dwell time gradient restriction, HIPO: hybrid inverse planning optimization

Contour	Volume	Surface	Density	% on Surface
HR-CTV	1,000	1,186	10	100
Bladder				90
Rectum				90
Sigmoid				90
Bowel				90
Normal tissue				-
DTGR	0.0 – 1.0			

Plan evaluation

Evaluation of the Dose-Volume Histogram (DVH) Analysis

DVH analysis was conducted for each treatment plan, and statistical analysis was performed to identify potential differences between the treatment plans for each DVH parameter. IBM SPSS Statistics 2.5 Windows (IBM Corp., Armonk, NY) was used for statistical analysis, and the significance level was set at p < 0.05. The analyzed parameters were based on the GEC-ESTRO Committee guidelines [19]. For the HR-CTV, the analysis included the percentage of the volume enclosed by D100, D90, and 7 Gy. These values represent the minimum doses delivered to 100% and 90% of the HR-CTV volume, with V7 Gy calculated accordingly. Additionally, for the rectum, sigmoid colon, and bladder, we calculated D2cc, which represents the minimum dose received by 2 cc and the region irradiated with the highest dose. We also compared the volumes of V300, V200, V150, and V100 surrounded by 300%, 200%, 150%, and 100% of the prescribed dose (7 Gy).

Treatment Planning Indices

The dose homogeneity index (DHI) was measured as dose uniformity for the HR-CTV [21]. It is determined using Equation (1) as follows:

DHI = (TVDref -TV1.5ref)/TVDref (1)

where TVD ref and TV1.5ref are the 100% and 150% volume receiving at least the prescribed dose of the HR-CTV, respectively.

The conformity index (COIN), calculated using Equation (2), was used to represent the irradiated volume of the target and normal tissue [21]:

COIN = (TVref/TV) × (TVref/Vref) (2)

where TVref is the portion of the HR-CTV that received at least the prescribed dose, TV is the volume of the HR-CTV, and Vref is the total volume that received at least the prescribed dose. The DHI and COIN are 1.0 for an ideal case, the dose index of treatment planning was used to define the quality of the treatment plans, considering both the target coverage and degree of normal tissue irradiation.

Distribution of Dwell Time

The distribution of dwell time of inverse planning reported that there is a large variation in the dwell times [9,22]. Thus, the distribution of dwell time for all treatment fractions was evaluated by measuring the time required for the optimization of treatment planning (Manchester method, IPSA, HIPO, and HIPO + sampling point).

Optimize Time

The optimization times for all treatment fractions were evaluated by measuring the time required for the optimization of the treatment planning. The Manchester method optimization times were determined using the dose point optimization method for the dwell times of all sources. The IPSA, HIPO, and HIPO + sampling point optimization times were used to calculate the source placement and dwell time by specifying the target settings for multiple targets and OARs. The statistical difference was determined by Kruskal-Walli's test. Difference with P < 0.05 was considered significant.

## Results

Evaluation of DVH analysis

Table 5 summarizes the DVH parameters (mean ± standard deviation) of the HR-CTV and OARs for each treatment plan. For DTDC and DTGR = 0.0, the HR-CTV D90 was 7.15 ± 0.23, 7.64 ± 0.19, 7.08 ± 0.34, and 7.17 ± 0.32 Gy in the Manchester method, IPSA, HIPO, and HIPO + sampling points, respectively. For DTDC and DTGR = 0.5, the HR-CTV D90 was 7.15 ± 0.23, 6.59 ± 0.83, 6.82 ± 0.41, and 6.93 ± 0.41 Gy in the Manchester method, IPSA, HIPO, and HIPO + sampling points, respectively. Finally, for DTDC and DTGR = 1.0, the HR-CTV D90 was 7.15 ± 0.23, 5.21 ± 1.06, 6.76 ± 0.45, and 6.83 ± 0.44 Gy in the Manchester method, IPSA, HIPO, and HIPO + sampling points, respectively.

**Table 5:** Summary of DVH parameters. IPSA: inverse planning simulated annealing, HIPO: hybrid inverse planning optimization, HR-CTV: high-risk clinical target volume, OAR: organ at risk, DVH: dose-volume histogram

Plan	Manchester	IPSA				HIPO				HIPO + sampling point		
	(m)	(i) 0.0	(i) 0.5	(i) 1.0		(h) 0.0	(h) 0.5		(h) 1.0	(hs) 0.0	(hs) 0.5	(hs) 1.0
Parameter	Mean ± SD	Mean ± SD				Mean ± SD				Mean ± SD		
HR-CTV												
D_90_ (Gy)	7.15 ± 0.23	7.64 ± 0.19	6.59 ± 0.83		5.21 ± 1.06	7.08 ± 0.34	6.82 ± 0.41	6.76 ± 0.45		7.17 ± 0.32	6.93 ± 0.41	6.83 ± 0.44
D_100 _(Gy)	3.22 ± 0.29	3.42 ± 0.29	2.41 ± 0.23		1.96 ± 0.31	2.96 ± 0.33	2.79 ± 0.33	2.71 ± 0.33		2.92 ± 0.46	2.87 ± 0.29	2.84 ± 0.38
V_7Gy_ (%)	90.6 ± 2.03	92.1 ± 3.14	81.4 ± 2.79		70.6 ± 2.85	88.9 ± 3.97	88.2 ± 4.12	86.8 ± 3.69		89.5 ± 2.29	88.4 ± 3.01	87.8 ± 1.99
OAR D_2cc _(Gy)												
Bladder	6.18 ± 0.37	6.20 ± 0.37	6.20 ± 0.38		5.98 ± 0.38	5.56 ± 0.55	5.65 ± 0.55	5.63 ± 0.55		5.58 ± 0.55	5.65 ± 0.69	5.65 ± 0.56
Rectum	5.39 ± 0.71	5.33 ± 0.68	5.24 ± 0.67		5.15 ± 0.62	5.18 ± 0.70	5.16 ± 0.68	5.15 ± 0.67		5.23 ± 0.71	5.21 ± 0.69	5.21 ± 0.69
Small bowel	4.13 ± 0.98	3.76 ± 1.51	3.09 ± 0.94		2.97 ± 0.93	4.21 ± 1.35	3.75 ± 1.29	3.76 ± 1.29		3.81 ± 1.27	3.79 ± 1.31	3.80 ± 1.31
Volume (cm^3^)												
V_100_	94.6 ± 0.24	94.1 ± 1.29	86.1 ± 1.30		78.8 ± 5.72	90.7 ± 2.96	88.9 ± 3.34	88.3 ± 3.51		91.5 ± 2.80	89.6 ± 3.29	88.9 ± 3.45
V_150_	54.2 ± 3.37	52.1 ± 4.55	48.4 ± 3.68		42.7 ± 3.16	47.5 ± 1.15	46.8 ± 1.97	52.1 ± 4.60		52.1 ± 4.61	47.6 ± 3.29	47.3 ± 2.32
V_200_	40.5 ± 9.94	36.7 ± 3.74	34.3 ± 3.68		30.7 ± 3.11	31.0 ± 1.75	32.1 ± 1.97	36.7 ± 3.79		36.7 ± 3.80	32.8 ± 2.20	32.7 ± 1.87
V_300_	19.7 ± 5.13	16.6 ± 2.41	14.2 ± 1.85		12.9 ± 2.01	15.5 ± 1.48	14.9 ± 0.98	18.6 ± 2.46		18.6 ± 2.47	15.2 ± 1.06	15.3± 0.97

The DTDC and DTGR = 1.0, the D90 of the HR-CTV was significantly lower than that compared with the Manchester method. The D100 and V7 Gy of the HR-CTV were not significantly different from those of the other optimization methods. The OAR did not have a D2cc greater than 6.5 Gy with dose reservation in any of the treatment plans. The rectum and small bowel D2cc values were lower with the other optimization methods than with the Manchester method. Compared to the Manchester method, V300, V200, V150, and V100 were optimized by IPSA, HIPO, and HIPO + sampling points were not significantly different, and IPSA was the lowest on average (Table 6).

**Table 6:** Summary of DVH parameters. Statistical significance was set at P < 0.05. HR-CTV: high-risk clinical target volume, OAR: organ at risk, DVH: dose-volume histogram

Plan	(m) vs (i) 0.0 vs (h) 0.0 vs (hs) 0.0			(m) vs (i) 0.5 vs (h) 0.5 vs (hs) 0.5			(m) vs (i) 1.0 vs (h) 1.0 vs (hs) 1.0		
	(i) 0.0	(h) 0.0	(hs) 0.0	(i) 0.5	(h) 0.5	(hs) 0.5	(i) 1.0	(h) 1.0	(hs) 1.0
Parameter	P-value								
HR-CTV									
D_90_ (Gy)	0.12	0.39	0.59	0.10	0.12	0.16	< 0.05	< 0.05	< 0.05
D_100 _(Gy)	0.11	0.08	0.07	0.21	0.09	0.07	0.19	0.12	0.11
V_7Gy_ (%)	0.13	0.23	0.27	0.24	0.31	0.33	0.25	0.30	0.35
OAR D_2cc _(Gy)									
Bladder	0.38	0.37	0.31	0.21	0.20	0.19	0.17	0.19	0.22
Rectum	0.44	0.38	0.34	0.36	0.31	0.37	0.21	0.24	0.25
Small bowel	0.09	0.41	0.11	0.06	0.25	0.27	0.06	0.19	0.22
Volume (cm^3^)									
V_100_	0.21	0.33	0.31	0.31	0.33	0.32	0.06	0.37	0.37
V_150_	0.25	0.38	0.31	0.36	0.39	0.30	0.23	0.26	0.24
V_200_	0.16	0.33	0.21	0.11	0.42	0.38	0.19	0.07	0.17
V_300_	0.28	0.16	0.26	0.35	0.09	0.19	0.09	0.42	0.11

Treatment planning evaluation indices

**Figure 2:**
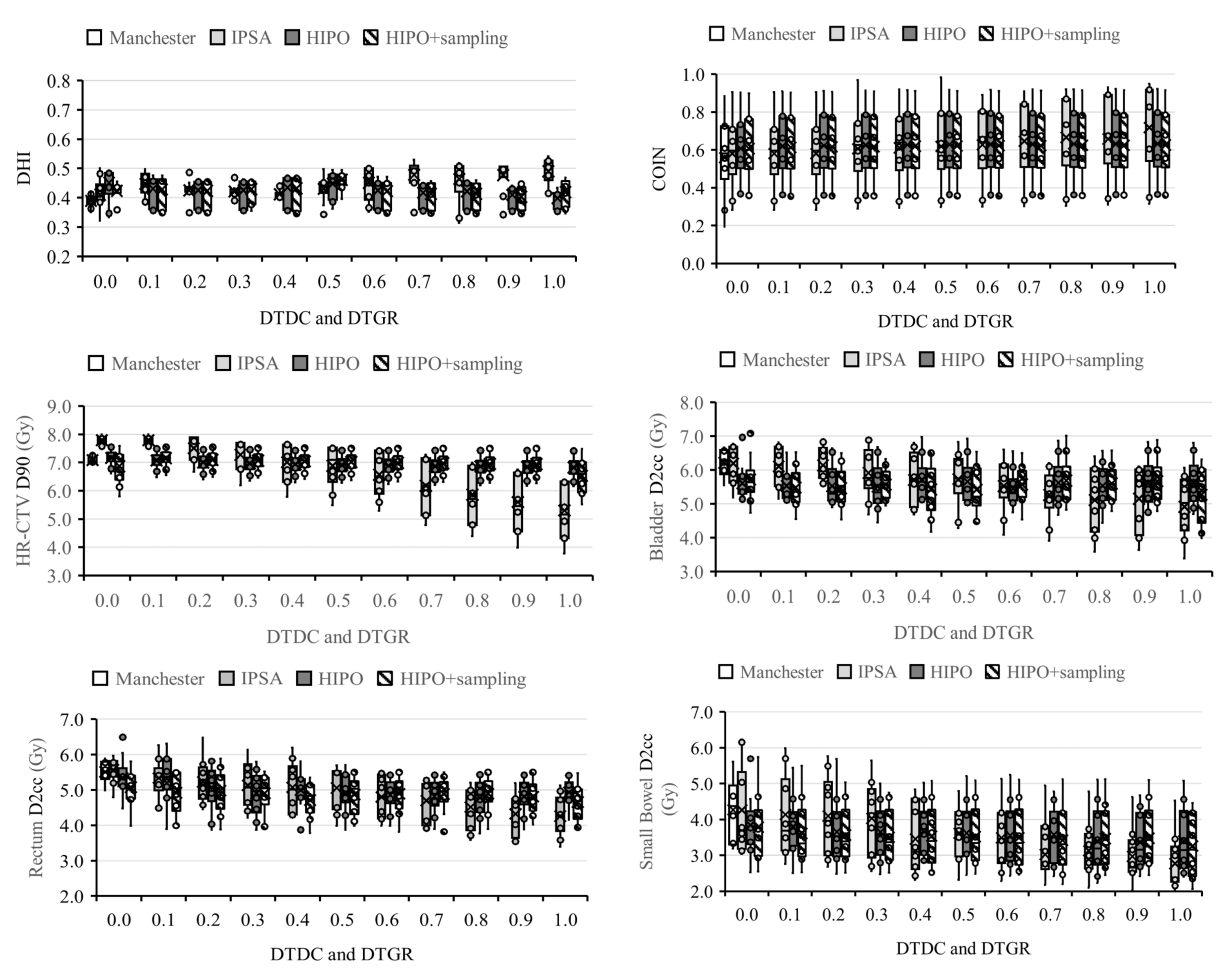
Box plots of the dosimetric parameters for the optimization methods. IPSA: inverse planning simulated annealing, HIPO: hybrid inverse planning optimization, DTDC: dwell time deviation constraint, DTGR: dwell time gradient restriction

Figure 2 illustrates the treatment plan indicators calculated for each treatment plan as box plots. The average DHI value was below 0.5, regardless of the DTDC and DTGR parameters. The average COIN values were above 0.6, regardless of the DTDC and DTGR parameters. The IPSA of HR-CTV D90 and OAR D2cc tended to decrease as the DTDC values increased. In contrast, no change was observed in the HIPO and HIPO + sampling points of HR-CTV D90 and OAR D2cc, regardless of the DTGR parameter values.

Distribution of dwell time

**Figure 3:**
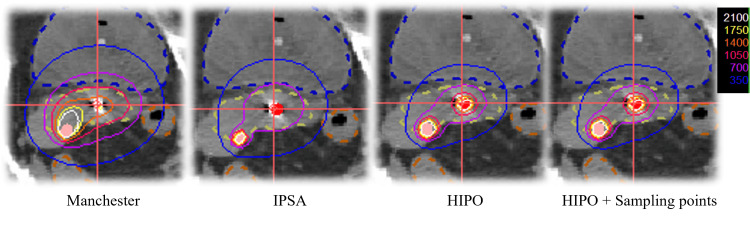
Dose distribution for each technique. Axial dose distributions made by each method are shown, respectively. A dotted line indicates HR-CTV (yellow). IPSA: inverse planning simulated annealing, HIPO: hybrid inverse planning optimization

Figure 3 illustrates the dose distribution in the axial plane for each treatment plan examined at this time (DTDC, DTGR = 0.5). The dose distributions for IPSA, HIPO, and HIPO+ sampling points were steeper than those for the Manchester method.

**Figure 4:**
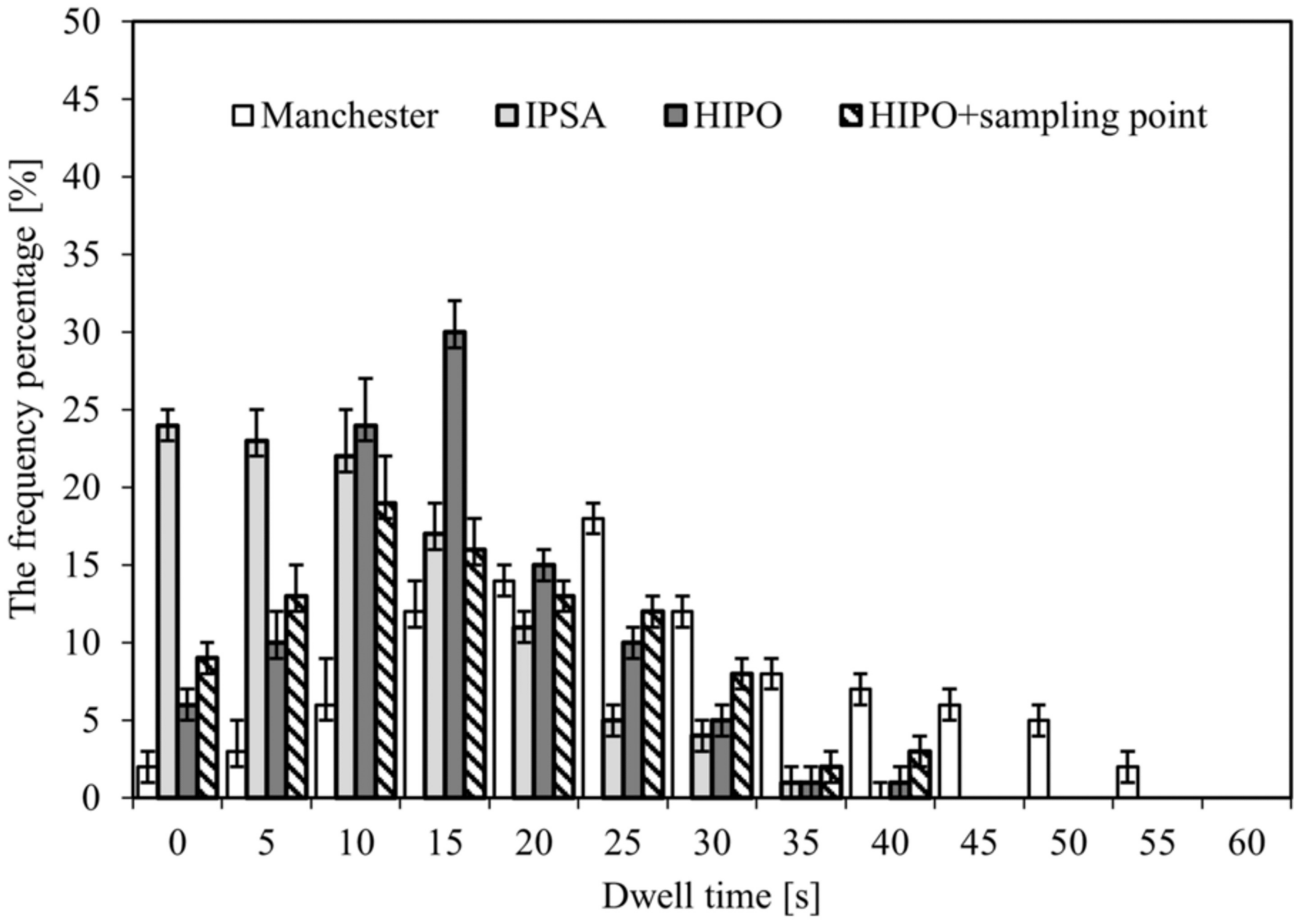
Distribution of dwell times due to different optimization methods. IPSA: inverse planning simulated annealing, HIPO: hybrid inverse planning optimization

Figure 4 illustrates the distribution of dwell times due to different optimization methods. The mean ± standard deviation of the dwell times was 24.3±5.9 sec, 5.9±14.4 sec, 8.8±9.4 sec, and 15.2±10.4 sec for the Manchester method, IPSA, HIPO, and HIPO + sampling points, respectively. IPSA had shorter dwell times than the other inverse planning methods.

Optimization times

**Figure 5:**
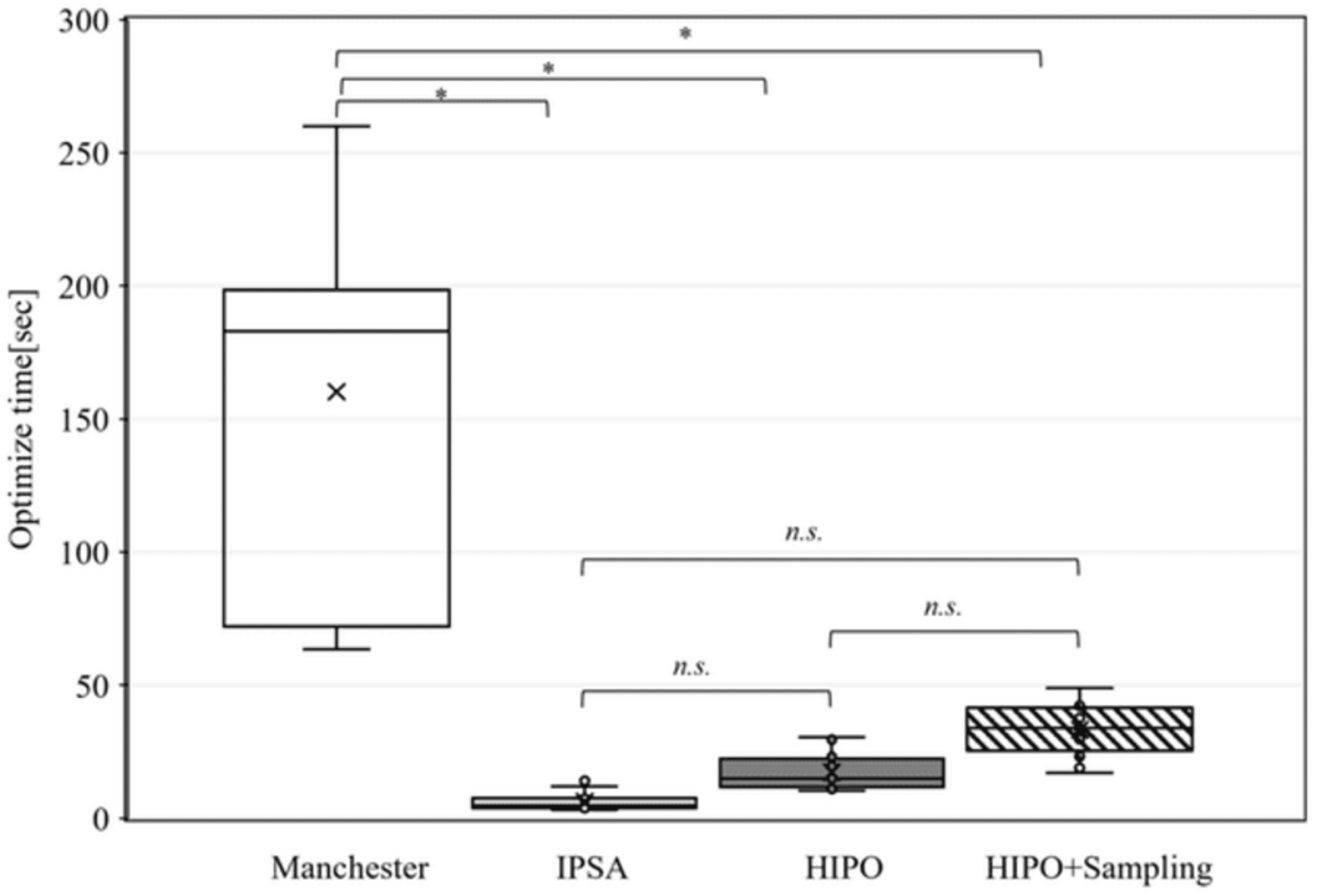
Optimization times due to different optimization methods. *Kruskal-Walli's test; P<0.05 IPSA: inverse planning simulated annealing, HIPO: hybrid inverse planning optimization

Figure 5 illustrates the optimization times for the different optimization methods. The mean ± standard deviation of the dwell times was 180.9±38.9 sec, 4.5±2.9 sec, 23.8±5.7 sec, and 40.1±14.6 sec for the Manchester method, IPSA, HIPO, and HIPO + sampling points, respectively. The optimization time was significantly shorter in the order of IPSA, HIPO, and HIPO + sampling points compared to the Manchester method.

## Discussion

In this study, we investigated three methods for cervical cancer BT: the IPSA algorithm, the HIPO algorithm, and the HIPO + sampling, and examined their clinical usefulness. Our results revealed that inverse planning could demonstrate equivalent DVH parameters in a short time using the Manchester method and optimization using IPSA, HIPO, and HIPO + sampling. In addition, the influence of the DTDC and DTGR parameters was revealed in clinical cases.

For DTDC and DTGR = 0.0, the D90 of HR-CTV in IPSA was significantly higher than those of the other methods. Also, for DTDC and DTGR = 0.5 and 1.0, IPSA was significantly lower than those of the other methods. The treatment plan indicators calculated from each treatment plan, the average DHI and COIN values, were almost constant regardless of DTDC and DTGR parameters. IPSA and HIPO + sampling points of D90 of HR-CTV and D2cc of OAR tended to decrease as DTDC and DTGR parameter values increased. On the other hand, no change was observed in HIPO for D90 of HR-CTV and D2cc of OARs regardless of the DTGR parameter values.

In an IPSA optimization, the parameter DTDC restricts the difference in dwell times between adjacent dwell positions within each catheter. The use of the DTDC avoids the presence of isolated positions with extremely large dwell times. However, studies have shown that a high DTDC value may work against the target coverage and OAR sparing [23]. This study showed a tendency for DTDC parameter values to increase, and HR-CTV D90 and OAR D2cc to decrease. In addition, it usually provided plans that are characterized by short dwell times between each catheter (Figure 4). This behavior can lead to large delivery errors in the case of catheter movement by under- and overdosing the target or the OARs. For plans obtained with IPSA, to avoid such hot spots, it is recommended to review the dose distribution for users to manually limit large dwell times and then proceed to a final dose distribution using graphical optimization. All these steps result in shorter optimization times than other techniques, allow for repeatable treatment planning, and increase the reproducibility and robustness of the treatment planning process. The DTGR is a modulation restriction parameter for HIPO that restricts large fluctuations between dwell times in neighboring dwell positions. However, in this study, the adverse effects on target coverage and OAR tended to be minimal regardless of the DTGR parameter setting. In this study, HIPO and HIPO+ sampling points also showed similar trends in mean DHI and mean CI, HR-CTV D90, and OAR D2cc.

The distribution of dwell times due to different optimization methods was compared to that of the Manchester method, and the IPSA, HIPO, and HIPO+ sampling points tended to have shorter median residence times and greater variation. The IPSA has very short or long residence times and an inhomogeneous distribution. Therefore, considering the effects of the transit dose [24], it may be necessary to manually modify the dose before it is implemented for treatment. Compared to IPSA, HIPO and HIPO+ sampling points produced a smoother dwell time distribution, which may result in more clinically desirable dose distributions (Figure 3). HIPO was superior at eliminating high-dose regions from normal tissues. However, the dwell position and time must be manually set. The settings of the sampling points for dose optimization are fully automated in IPSA but manually adjustable in HIPO. The optimization times due to the different optimization methods were significantly shorter in the order of IPSA, HIPO, and HIPO + sampling points compared to the Manchester method. These IPSA cases showed large fluctuations between dwell times in neighboring dwell positions, resulting in some dwell positions having very long times, whereas others had short times or were empty. However, the IPSA optimization calculations can be easily repeated, and the distribution can be created efficiently.

The IPSA and HIPO have a trade-off relationship. This was caused by the modulation restrictions of the dwell times by the use of the DTDC and DTDG parameters. This shows that the residence times obtained using the optimization calculations form a wavy distribution, and it is important to ensure that the adjacent residence time changes are continuous and smooth.

One of the limitations of this study regarding the optimization method using IC/IS is that a small number of needles were used to evaluate cervical cancer. This uncertainty may be greater than that for many needles or other cases. In the future, we would like to consider optimization methods for precision medicine.

## Conclusions

IC/IS-BT benefits from the use of inverse planning performed by optimization algorithms. In this study, the widely used Manchester method was compared with IPSA, HIPO, and HIPO + sampling points recently implemented in the TPS. 

The DVH parameters and dose distribution shape provided by the optimization parameters were evaluated for each irradiation session of IC/IS-BT, and their relationship with each optimization parameter and optimization time was clarified. It was suggested that IPSA may result in extreme source dwell positions and dwell times, particularly with IC/IS-BT.
